# Oviposition Preference of the American Hoverfly, *Eupeodes americanus*, between Banker Plants and Target Crops

**DOI:** 10.3390/insects14030295

**Published:** 2023-03-19

**Authors:** Noémie Gonzalez, Arlette Fauteux, Jean-Christophe Louis, Rosemarije Buitenhuis, Eric Lucas

**Affiliations:** 1 Laboratoire de Lutte Biologique, Département des Sciences Biologiques, Université du Québec à Montréal (UQAM), Succursale Centre-Ville, P.O. Box 8888, Montréal, QC H3C 3P8, Canada; 2 Vineland Research and Innovation Center, 4890 Victoria Ave. N., P.O. Box 4000, Vineland Station, ON L0R 2E0, Canada

**Keywords:** aphidophagous hoverflies, Syrphidae, biocontrol, *Aphis gossypii*, *Myzus persicae*, *Rhopalosiphum padi*, greenhouse crops

## Abstract

**Simple Summary:**

Oviposition preference of aphidophagous hoverflies is a key factor in the biological control of aphids, especially when using banker plant systems. In this laboratory study, the oviposition preferences of *Eupeodes americanus* were evaluated in two-choice experiments with different plant/aphid systems. The results show that the finger millet banker plant may be more suitable for the control of *A. gossypii* in cucumber crops and the barley banker plant for the control of *M. persicae* in pepper crops. Finally, this study demonstrates that the oviposition of *E. americanus* can be adequate for the control of aphids in a mixed crop of cucumber and pepper. Future semifield or field studies are needed to confirm these recommendations.

**Abstract:**

Assessing the oviposition preferences of predatory hoverflies is a key factor in the prediction of the success of these biological control agents against aphids in greenhouses, especially when using banker plant systems or in mixed-crop contexts. In this study, two aspects of the oviposition preferences of the American hoverfly, *Eupeodes americanus* (Wiedemann, 1830) (Diptera: Syrphidae), were evaluated. Firstly, the preference between the banker plant and the target crop was evaluated for three banker plant species (barley, finger millet, or corn) and two target crops (cucumber or pepper). Secondly, the preference between the same two target crops was assessed. Female oviposition preferences were evaluated via two-choice experiments using different plant/aphid systems. The results showed that, for the cucumber crops, the species of banker plant used drastically influenced the oviposition preference of the hoverfly, with a preference for barley over cucumber, cucumber over finger millet, and no preference between corn and cucumber. Unlike cucumber, when used with pepper, barley engendered a preference for the target crop. We conclude that the barley banker plant could be adequate for aphid control in pepper but not in cucumber crops. In a mixed-crop context, the American hoverfly had no preference between cucumber and pepper, which means it has the potential to protect both crops in a mixed-crop greenhouse context. This study shows that the banker plant system should be carefully chosen according to the crops/aphids present in the greenhouse to optimize the impact of the hoverfly as a biocontrol agent. Further work is required to confirm this choice of banker plant in semifield or field testing.

## 1. Introduction

Aphids are major greenhouse pests [1,2]. Their management used to be mainly achieved via chemical control, but nowadays, the use of pesticides can be problematic because aphids may become resistant to several active ingredients and because of their harmful effects [1,3,4]. As an alternative, more and more growers implement integrated pest management (IPM). In temperate climates, biological aphid control strategies generally include inundative releases of parasitoids of the genus *Aphidius* combined with predators like the aphid midge, *Aphidoletes aphidimyza* Rondani, 1847 (Diptera: Cecidomyiidae) [2]. Predatory hoverflies, such as *Sphaerophoria rueppellii*, *Episyrphus balteatus*, and *Eupeodes corollae*, are also used in greenhouses in Europe [5,6,7,8]. However, those biological control agents have variable efficacy against aphids across crops and are not always reliable as stand-alone treatments [9,10,11]. Moreover, this method can be costly as a preventive strategy, especially when using biological control agents with a short life cycle since they need to be introduced too often [12,13]. Therefore, aphids remain a major concern in several greenhouse crops [2]. For example, the melon aphid, *Aphis gossypii* Glover 1877 (Hemiptera: Aphididae), is today still one of the most important pests limiting the production of cucumber (*Cucumis sativus* L.) in many countries [14,15,16,17,18]. The development of new biological control methods is, therefore, necessary to improve aphid control and prevent crop losses.

The main problem associated with the biological control of aphids as a curative method is the delayed action of natural enemies [12,19]. Even when aphid colonies are found early, the delay between detection, the introduction of biological control agents, and the time required for them to take effect often allow pest populations to increase beyond the economic threshold [12]. Among other methods, banker plants could constitute a potential solution as they ensure the constant presence of biological control agents in the crop by providing an alternative food source and oviposition sites even before the arrival of pests [20,21]. One possible drawback of banker plants is that they may act as a sink for the biocontrol agents and, thus, divert them from the target crop [20,21]. The success of banker plants depends, then, largely on balance between the quality of the banker prey and the oviposition preference of the predator for the target prey. The biological control agent must be able to develop and reproduce on the banker plant but choose to leave it, at least partially, when pests invade the crop [20,22]. Therefore, when evaluating the efficacy of a biocontrol agent used in a banker plant system, it is necessary to investigate its oviposition behavior and, more specifically, its preference among all the plants and aphid species involved [20]. Moreover, in greenhouse production, two or three aphid species are often present at the same time. For example, intercropping integrates two crops or more under the same greenhouse [23,24,25], creating a similar scenario where oviposition behavior is an important factor for biological control efficacy.

Determining the optimal use of banker plants is also very important for new biocontrol agents. Predatory flies of the Syrphidae family (Diptera) generally exhibit characteristics that predispose them to be successful biological control agents. For example, they tend to have a high voracity, a good flight and searching ability, and a high fecundity [26,27]. The American hoverfly, *Eupeodes americanus* (Wiedemann, 1830) (Diptera: Syrphidae), which, at the aphidophagous larval stage and a pollinator adult stage, shows great potential at controlling aphids. Previous research at the Biocontrol laboratory of Université du Québec à Montréal (UQAM) on the American hoverfly has shown that this species is active at low temperatures, e.g., for flight, oviposition, and predation activities [28], which enables it to efficiently control the foxglove aphid, *Aulacorthum solani* (Kaltenbach 1843) (Hemiptera: Aphididae) in Canadian greenhouse crops [29]. Furthermore, *E. americanus* has a longer larval development time (i.e., predacious stage) and longer adult longevity compared to the commercially available aphidophagous predator *A. aphidimyza* [30]. However, knowing that hoverfly larvae do not disperse very well, their control depends largely on the dispersion and oviposition of the females [27].

Hoverfly oviposition preference is influenced by numerous factors, such as predation risk and the presence of intraspecific or interspecific competitors [31,32,33,34,35]. However, the most important factors are usually aphid species, nutritional quality, and density [35,36,37]. For example, the female hoverflies of *Episyrphus balteatus* (De Geer, 1776), (Diptera: Syrphidae) prefer to lay their eggs in colonies on the green peach aphid, *Myzus persicae* (Sulzer, 1776) (Hemiptera: Aphididae) and the pea aphid, *Acyrthosiphon pisum* (Harris, 1773) (Hemiptera: Aphididae) rather than in colonies on the vetch aphid, *Megoura viciae* Buckton, 1876 (Hemiptera: Aphididae) on broad beans (*Vicia faba* L.) [38]. Another factor influencing the oviposition preference of syrphid females is the host plant species, both in terms of physical traits and chemicals emitted by the plant [35,37]. According to Vanhaelen et al. 2001 [38], *E. balteatus* prefers white mustard (*Sinapis alba* L.) to rapeseed (*Brassica napus* L.) and broad beans.

The present study aims to evaluate the oviposition behavior of the American hoverfly in common greenhouse contexts in temperate regions, that is, cucumber or pepper (*Capsicum annuum* (L.)) crops, with or without banker plants. Both of those crops are affected by major pests: the melon aphid on cucumber and the green peach aphid on pepper [39,40]. Three species of banker plants were evaluated in this study: barley (*Hordeum vulgare* L.), corn (*Zea mays* L.), and finger millet (*Eleusine coracana* Gaert). Those plant species were selected because either they are commonly used, such as barley, or have already demonstrated their efficiency in previous studies, such as corn and finger millet [12,20,21,29,41,42]. They are also well suited for the experiment since they are all used with the bird cherry-oat aphid, *Rhopalosiphum padi* L. (Hemiptera: Aphididae), as prey.

Our first objective was to evaluate if the oviposition preferences of the American hoverfly are suitable for the control of the melon aphid on cucumber using three banker plant species. For that, we verified if the American hoverfly prefers laying eggs on cucumber rather than on the banker plants. We also verified if some banker plant species were more suitable than others, i.e., leading to a larger proportion of eggs laid on the focal crop and a higher total number of eggs.

Our second objective was to verify that the oviposition preference of the American hoverfly is suitable for the control of the green peach aphid on pepper using the barley banker plant (being the most commonly used), expecting that, proportionally, more eggs are laid on pepper than on barley. We also verified if that banker plant was more appropriate for this crop than for cucumber, i.e., leading to a larger proportion of eggs laid on the focal crop and a higher total number of eggs.

Finally, our third objective was to verify if the oviposition behavior of the American hoverfly was suitable for the simultaneous control of both the melon aphid on cucumber and the green peach aphid on pepper in a mixed crop greenhouse context, expecting an equal proportion of eggs laid on both focal crops.

## 2. Materials and Methods

### 2.1. Plants

The crop plants used during the experiment were pepper (Solanaceae) (cv. hybrid Aristotle X3R, Norseco, Laval, Canada) and cucumber (Cucurbitaceae) (cv. hybrid Speedway, Norseco). The banker plants used were barley (Sollio Agriculture, Quebec, Canada), finger millet (Snake River Seed Cooperative, USA), and corn (Sollio agriculture). All plants were sown and grown in the greenhouses of UQAM at 25 °C during the day, 19 °C at night, and 60% relative humidity (RH) and 16:8 (L:D) under high-pressure sodium lamps. Seedlings of cucumber, pepper, and corn were transplanted in plastic pots (9 × 9 cm). The number of barley and finger millet seeds was constant between the replicates (identical volume planted in plastic pots (9 × 9 cm)). The plugs and substrate used were a humus-content potting mix enriched with compost (Garden soil, Fafard, Agawam, USA). The plants were watered as needed and provided weekly with a fertilizer (20–20–20 NPK). No chemical insecticides were applied to the plants. All species used during the experiment had a vegetative growth phenological stage. Cucumber had 4 leaves, pepper had 6–7 leaves, and corn had 4–5 leaves. Barley and finger millet were approximately 15 cm in height.

### 2.2. Insect Rearing

All insect colonies were kept at UQAM in the Biocontrol laboratory. *Aphis gossypii* were reared on cucumber and *M. persicae* on pepper in a 35 × 35 × 35 cm cage kept in a growth chamber at 24 °C, with a 16:8 (L:D) photoperiod and 70% RH. Wild adults of *E. americanus* were collected on *Phlox* sp. (L.) in Sainte-Agathe-de-Lotbinière (N 46°23′726″ W 71°21′446″), Québec, Canada, in 2014. Hoverfly colonies were refreshed yearly with new wild individuals. American hoverfly rearing was done as described in Bellefeuille et al. (2019) [28] except for the oviposition method. In the present case, four broad bean plants (*Vicia faba* (L.)) infested with pea aphids, *Acyrthosiphon pisum* (Harris, 1776) (Hemiptera: Aphididae) were placed in the center of the cage to allow females to lay eggs after mating. The larvae were transferred to barley plants infested with the bird cherry-oat aphid. When needed, *R. padi* were transferred onto finger millet or corn before using them for the experiment.

### 2.3. Objective 1: Suitability of Three Banker Plant Species to Control A. gossypii on Cucumber

**Figure 1:**
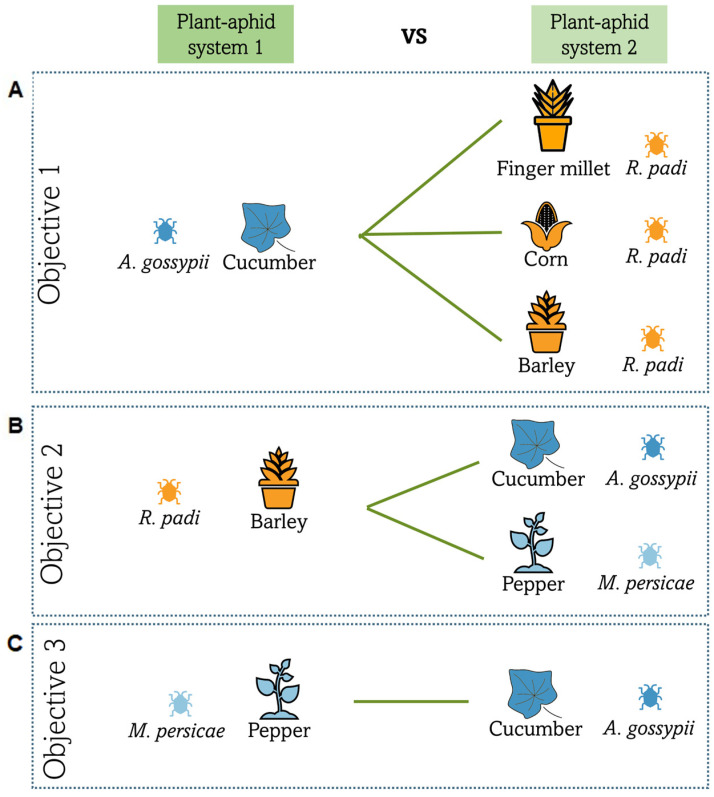
Composition of the different choice trials as plant/aphid systems. Banker plants are represented in orange, cucumber in blue, and pepper in light blue.

To evaluate the suitability of the three banker plants, the oviposition preferences of *E. americanus* between each banker plant and *A. gossypii* on cucumber were verified. Three different choice trials (with barley, finger millet, and corn) were performed (Figure 1A).

**Figure 2:**
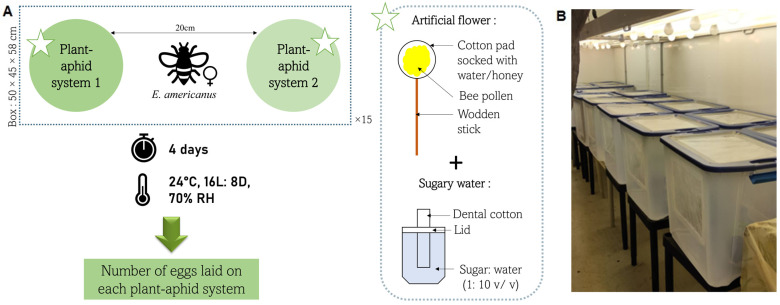
(**A**) Experimental design of the choice trial used to determine the oviposition preference of *E. americanus* between two different plant/aphid systems; (**B**) plastic boxes used to conduct the choice trials and placed in a Conviron growth chamber.

Each choice trial was carried out in a 50 × 45 × 58 cm transparent plastic box with a muslin-screened lid, and two 20 × 20 cm screened windows (Figure 2B). One banker plant with *R. padi* (alternative prey) was placed at one end of the box, and one cucumber plant with *A. gossypii* (focal crop and pest) was placed at the opposite end of the box (Figure 2A). A total of 100 aphids of mixed developmental stages were placed on each plant in the box. Such a high number of aphids was chosen to better study oviposition since hoverflies prefer to lay eggs on plants with high densities of aphids [26]. Each plant was provided with an artificial flower and a mixture of sugar:water (1:10 *v*/*v*) in a small cup with a roll of dental cotton sticking out of the lid for feeding the adult hoverflies. The artificial flower was made of a wooden stick with a cotton pad at its end, soaked in a mixture of water and honey, and covered with bee pollen (Miel Gauvin Inc., Saint-Hyacinthe, Canada) (Figure 2A). For each replicate, a one-week-old female hoverfly was released in the middle of the plastic box between the two plant/aphid systems (Figure 2A). The test lasted four days, during which time the box was placed in a Conviron growth chamber at 25 °C, 16:8 (L:D) photoperiod, and 50% RH (Figure 2B). After four days, the eggs laid were counted on each plant. In the case of barley and finger millet, the plants were cut at the base of each stem to ensure egg count accuracy. Fifteen replicates were performed per choice trial. The quality of the aphid colonies was checked, and the replicates in which the aphids did not develop correctly (abundance less than the initial individuals), were not considered.

Before their introduction to the experiments, female hoverflies that newly emerged were put together in a screen cage measuring 30 × 30 × 60 cm for one week at a ratio of two males to three females. The hoverflies were left in groups so that each female copulated with several males, which reduced the chances of using an unfertilized female for a test in the event of a dysfunctional male. They were fed with one artificial flower and sugary water, as described above. One broad bean plant infested with pea aphids was also placed in the center of the cage because the presence of an oviposition stimulus was proven to be necessary for hoverflies to lay fertile eggs and avoid the resorption of eggs [43,44].

### 2.4. Objective 2: Suitability of Barley Banker Plant Species to Control M. persicae on Pepper and Comparison with A. gossypii on Cucumber

To evaluate the suitability of the barley banker plant for the control of *M. persicae* on pepper, the oviposition preferences of *E. americanus* between those two plant/aphid systems were verified in a choice trial (Figure 1B). The same methods as for objective 1 were used. The results were compared with the choice trial realized for objective 1 between barley banker plant and *A. gossypii* on cucumber.

### 2.5. Objective 3: Suitability of the American Hoverfly to Control Aphids in a Mixed Crop Greenhouse Context

To evaluate the suitability of *E. americanus* in mixed crops, its oviposition preference between *M. persicae* on pepper and *A. gossypii* on cucumber was verified in a choice trial (Figure 1C). The same method as for objective 1 was used.

### 2.6. Data Analysis

Statistical analyses were carried out with R 4.0.5 software. For all the experiments, the normality and homoscedasticity of the residuals were verified with Shapiro–Wilk tests (*p* > 0.05) and the diagnostic plots were inspected (residuals vs. fitted, normal QQ plot, scale location, constant leverage). If they could not be obtained, even after square root, log, or inverse transformations, nonparametric tests were used. For each test, the significance level was set at alpha = 0.05. Throughout the manuscript, the sample size (n) is defined as the number of individuals or observations included in a statistical analysis.

For the first objective, the difference between the proportion of eggs laid by females on cucumber/*A. gossypii* and on the three banker plant systems was analyzed with nonparametric paired Wilcoxon tests or *t*-tests, depending on the normality and homoscedasticity of the residuals. Afterward, within each choice trial, the number of eggs laid by females on the target crop cucumber (and in total) were, respectively, square root- and log-transformed. The impact of the banker plant system (corn, barley, or finger millet with *R. padi*) on those two parameters was tested by one-way analysis of variance (ANOVAs). Posthoc Tukey’s HSD tests were then performed to identify which banker plant systems engendered a significantly different number of laid eggs in total and on the focal crop.

For objective 2, the difference between the proportion of eggs laid on pepper/*M. persicae* and on barley/*R. padi* was tested by a paired *t*-test. To verify if the barley banker plant was more adapted to the pepper or cucumber crops, the differences in the number of eggs laid during those two choice trials were analyzed (barley vs. pepper and barley vs. cucumber). The number of eggs laid on the focal crop (pepper or cucumber) was compared using a nonparametric paired Wilcoxon test. The total number of eggs laid in those choice trials (on the banker plant and on the focal crop) was compared with a *t*-test.

For objective 3, the difference between the proportion of eggs laid by females on the pepper/*M. persicae* and cucumber/*A. gossypii* crop systems was analyzed with a nonparametric paired Wilcoxon test.

## 3. Results

### 3.1. Objective 1: Suitability of Three Banker Plant Species to Control A. gossypii on Cucumber

**Figure 3:**
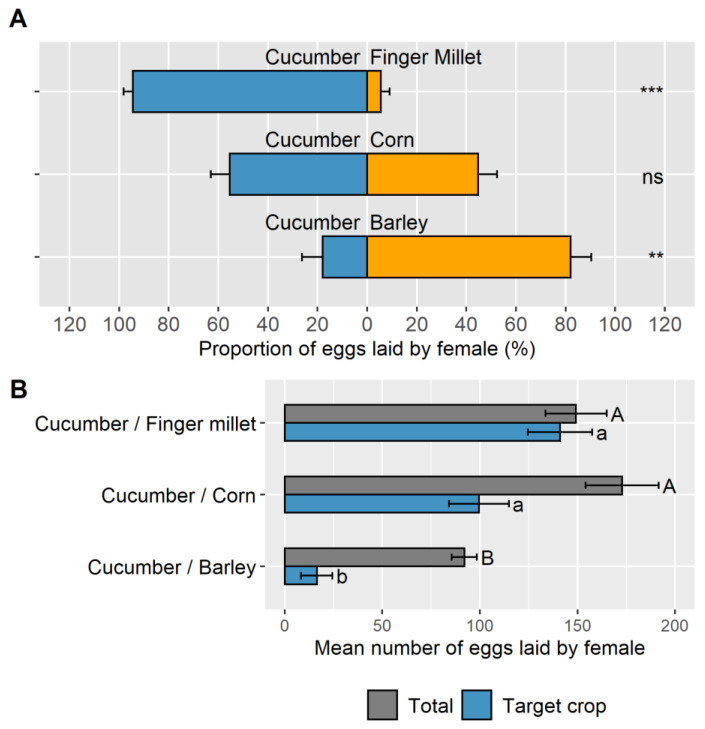
(**A**) Proportion of eggs laid by *E. americanus* between cucumber and the three banker plant systems. Significant differences between plant-aphid systems are shown by asterisks (alpha = 0.05, paired Wilcoxon test or *t*-test). The following significance code is taken into account: “***” corresponds to a *p*-value under 0.001, “**” *p*-value between 0.001 and 0.01. Beyond that, the *p*-values are codified with “ns”. The bars represent the mean ± SE; (**B**) number of eggs laid by *E. americanus* in choice trials involving cucumber and three banker plant systems. The letters indicate significant differences, with an alpha = 0.05. Uppercase letters indicate differences between the total number of eggs laid in 4 days (ANOVA followed by Tukey’s HSD test). Lowercase letters indicate differences between the number of eggs laid on the target crop (ANOVA followed by Tukey’s HSD test). The bars represent the mean ± SE.

Concerning the three choice trials (cucumber/*A. gossypii* and banker plant (barley, finger millet, and corn/*R. padi*), the females showed statistically significant oviposition preferences in two out of the three trials (Figure 3A). The female hoverflies laid a significantly larger proportion of eggs on the barley banker plant than on cucumber (respectively, 82.0 ± 8.0% and 18.0 ± 8.3%; n = 30, V = 8, and *p* = 0.003). Moreover, in some cases, the female chose to lay all her eggs only on one of the two plants. In this trial, 46.6% of females laid all their eggs only on barley. In contrast, the female hoverflies laid a significantly higher proportion of eggs on cucumber than on the finger millet banker plant (respectively, 94.5 ± 3.6% and 5.5 ± 3.6%; n = 30, V = 120, and *p* < 0.001) and 53.3% of females laid all their eggs only on cucumber. Finally, the female hoverflies had no oviposition preference between the cucumber and the corn banker plant (respectively, 55.4 ± 7.7% and 44,7 ± 7.7% of eggs laid on each plant/aphid system; n = 30, t = 0.653, df = 14, and *p* = 0.524) but only 6.6% of females laid all their eggs only on corn.

All females laid between 92.0 ± 6.4 and 173.0 ± 18.7 eggs in total during the trials (Figure 3B). This total number of eggs laid (per female) varied significantly according to the banker plant species vs. cucumber (n = 45, F = 10.15, df = 2, *p* < 0.001). Females laid 38 to 47% fewer eggs in the choice trial between cucumber and barley. The difference was significant compared to the choice trials between cucumber and finger millet or corn (respectively, *p* = 0.007 and *p* < 0.001) (Figure 3B).

Similarly, in those choice trials, the number of eggs laid on cucumber varied significantly, depending on which banker plant system it was paired with (n = 45, F = 28.82, df = 2, *p* < 0.001) (Figure 3B). The number of eggs laid on the target crop, cucumber, in the presence of barley as a banker plant was significantly lower than with finger millet by 88% (*p* < 0.001) and corn by 83% (*p* < 0.001) The number of eggs laid on cucumber with finger millet or corn as the banker plants was not significantly different (*p* = 0.122).

### 3.2. Objective 2: Suitability of Barley Banker Plant Species to Control M. persicae on Pepper and Comparison with A. gossypii on Cucumber

**Figure 4:**
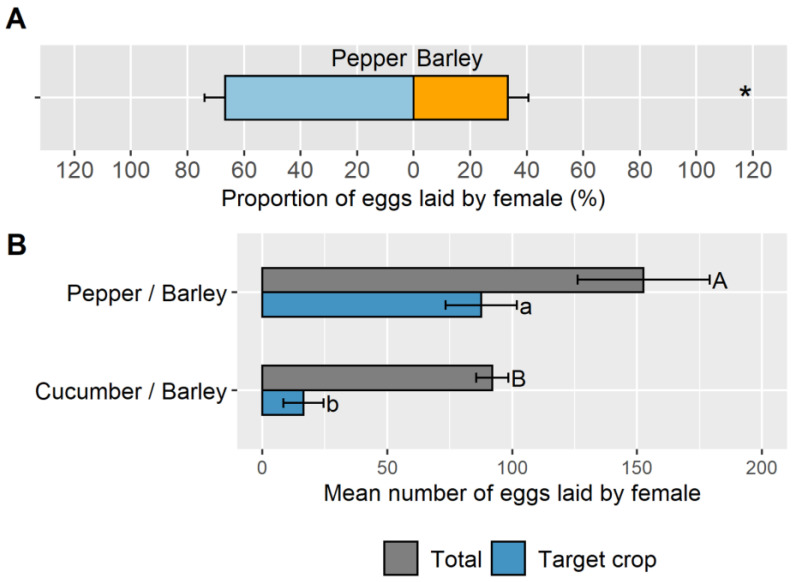
(**A**) Proportion of eggs laid by *E. americanus* between pepper and barley banker plant system. Significant differences between plant-aphid systems are shown by asterisks (alpha = 0.05, *t*-test). The following significance code is taken into account: “*” corresponds to a *p*-value between 0.01 and 0.05. Beyond that, the *p*-values are codified with “ns”. The bars represent the mean ± SE; (**B**) number of eggs laid by *E. americanus* in choice trials involving barley banker plant system and two major crops (cucumber and pepper). The letters indicate significant differences, with an alpha = 0.05. Uppercase letters indicate differences between the total number of eggs laid in 4 days (*t*-test). Lowercase letters indicate differences between the number of eggs laid on the target crop (paired Wilcoxon test). The bars represent the mean ± SE.

In the choice trial with pepper/*M. persicae* and the barley/*R. padi* banker plant system, the female hoverflies laid a significantly larger proportion of eggs on pepper compared to barley (Figure 4A) (respectively, 66.7 ± 7.3% and 33.3 ± 7.3%, n = 30, t = −2.372, df = 14, and *p* = 0.032), and 13.3% of females laid all their eggs only on pepper.

When comparing this trial with the choice trial between cucumber and barley (objective 1), the total number of eggs laid per female was significantly 66% higher in the choice trial with pepper than in the one with cucumber (n = 30, t = 2.2234, df = 15.647, and *p* = 0.041) (Figure 4B). Moreover, the number of eggs laid on the focal crop was significantly higher on a scale of 8.45 times in the choice trial involving pepper than in the one involving cucumber (n = 30, W = 18, *p* < 0.001) (Figure 4B).

### 3.3. Objective 3: Suitability of the American Hoverfly to Control Aphids in a Mixed Crop Greenhouse Context

**Figure 5:**
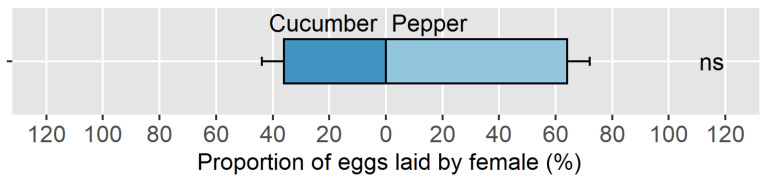
Proportion of eggs laid by *E. americanus* between pepper and cucumber. Significant differences between the plant-aphid systems are shown by asterisks (alpha = 0.05, paired Wilcoxon test). Beyond 0.05, the *p*-values are codified with “ns”. The bars represent the mean ± SE.

In the mixed crop choice trial, the female hoverflies had no oviposition preference between pepper/*M. persicae* and cucumber/*A. gossypii* (Figure 5) (respectively, 64.1 ± 7.9% and 35.9 ± 7.9% of eggs laid on each plant/aphid system; n = 30, V = 89, and *p* = 0.105), but 20.0% of the females laid all their eggs only on pepper.

## 4. Discussion

The oviposition preferences of the female hoverflies are influenced by numerous factors, including the host plant, aphid species, aphid colony size, the presence of intra- or interspecific competitors, female age, and the food resources for the adults [26]. All of these factors must be taken into account when establishing strategies for biological control using hoverflies against aphids. When using a banker plant system, a good biological control agent should prefer the target crop/prey combination to the banker plant to ensure the success of the biological control strategy [20,22,45]. Indeed, *E. americanus* should readily reproduce both on the banker plants and the target crop, and the newly emerged females from the banker plants should move quickly to the target crop [19,21,22,45]. In the present case, *E. americanus* should prefer *A. gossypii* on cucumber or *M. persicae* on pepper to *R. padi* on banker plants.

The results showed that when cucumber was the target crop, *E. americanus* oviposition preferences changed drastically depending on which banker plant was used. In this case, only the host plant species changed between choice trials since they were all carrying the same banker prey, *R. padi*. It is, therefore, the characteristics of these host plants which influenced the oviposition choice. *Eupeodes americanus* showed a significant preference for ovipositing on barley banker plant rather than on cucumber (82.0 ± 8.0% compared to 18.0 ± 8.3%), which could be due to the difference in leaf surface morphology. Indeed, hoverfly larvae and adults are negatively affected by plants with a high density of trichomes [26,46,47,48]. Sadeghi (2002) [49] and Almohamad et al. (2007) [37] proved that the oviposition preference of female syrphids is correlated with offspring performance on preferred host plants because the aphidophagous larvae have limited dispersal abilities [50,51,52]. For example, the oviposition of *E. balteatus* was lower on tomato cultivars, with a high density of trichomes, than on broad bean, *Vicia faba* L., which has a smooth surface [46]. This may explain the preference of the American hoverfly for the smooth-surfaced barley banker plant rather than the pubescent cucumber. Similarly, the American hoverfly did not have any oviposition preferences between cucumber and corn banker plant. This could be attributed to the fact that both plants have trichomes. Indeed, various studies have investigated the negative impact of corn leaf trichomes on insects and particularly on oviposition [53,54,55]. In parallel, the preference of *E. americanus* to oviposit on cucumber compared to the finger millet banker plant could be due to prey accessibility and availability [26,56]. Indeed, the dense architecture of the finger millet greatly reduces the prey’s accessibility to female hoverflies. Bird cherry-oat aphids, *R. padi*, at low density, are found hiding at the base of finger millet stems (personal observation), and the density of these stems constituting the banker plant left little access for the hoverflies.

The total number of eggs laid also varied between each choice trial. Indeed, our results showed that when combined with cucumber, both corn and finger millet induced a higher total number of eggs laid in each choice trial compared to barley. Further studies are needed to explain these results. For example, the differences in the quantity and composition of honeydew produced on the different banker plants could be an initial step to investigate since it can influence the number of eggs laid by hoverflies [51,57,58,59]. These results, and the strong preference for barley over cucumber, lead to a significantly lower mean number of eggs laid on the target crop than in the trials with corn and finger millet. In the context of the biological control of *A. gossypii* on cucumber by *E. americanus*, both finger millet and corn could constitute better banker plant systems than barley since they maximized both the oviposition of females and the number of eggs laid on the target crop cucumber. They also require less maintenance and have high longevity due to their resistance to hot greenhouse temperatures and high aphid abundance, especially compared to barley [12,19,41]. Nonetheless, finger millet was the only banker plant where *E. americanus* showed a strong negative preference compared to cucumber, which constitutes a good attribute for a banker plant (53.3% of females laid all their eggs only on cucumber). This is even more important in commercial greenhouses where the density of aphids, at least at the beginning of the infestation, will be higher on the banker plant than on the protected crop. This could redirect the preference towards the banker plant. Indeed, it has been proven that aphid density is also an important factor for hoverfly oviposition [26,52,59,60,61]. For this reason, finger millet is more appropriate than corn for the control of *A. gossypii* by *E. americanus* in cucumber, but future field or semifield studies are needed to confirm this conclusion.

The efficiency of a banker plant system also depends largely on the target crop it is used with. Indeed, a good banker plant for a specific target crop may not be appropriate in another greenhouse context. Our results concur with that since, contrary to cucumber, barley seems a more suitable banker plant for pepper. Indeed, females chose to oviposit preferentially on pepper over barley (66.7 ± 7.3% compared to 33.3 ± 7.3%). Moreover, both the total number of eggs laid and eggs laid on the target crop were higher in the pepper vs. barley trial than in the cucumber vs. barley trial. This higher total number of eggs laid can be explained by the different attributes of the pepper/*M. persicae* system, such as pepper plant morphology (smooth surface), aphid species preference, chemical cues, etc. [33,36,62]. Overall, the results suggest that the barley banker plant is more appropriate for the control of *M. persicae* in pepper crops than for the control of *A. gossypii* in cucumber crops. This highlights the great importance of choosing a specific banker plant system according to the target crop.

Finally, in the mixed crops, we expected the American hoverfly to prefer pepper because of its smoother surface than a pubescent cucumber, but our results showed that there was no oviposition preference between the two systems: pepper/*M. persicae* and cucumber/*A. gossypii*. From a biocontrol point of view, this means that the oviposition preference of *E. americanus* should not prevent the control of both pests in mixed crops. However, differences in the population growth rates between the two aphid species could be responsible for the observed results, and they should, therefore, be interpreted cautiously. Indeed, *A. gossypii* has a higher growth rate than *M. persicae* [2,63,64]; thus, the population density may have been different after the 4 days of the experiment. Since the oviposition of hoverflies is positively affected by higher aphid densities [26,52,59,60,61], the absence of preference may be due to changes in the relative densities of aphid species. Additionally, as demonstrated above, banker plants are not equally suitable for all crops, so in the case of mixed crops, care should be taken to find a banker plant that fits both crops.

Furthermore, in all the choice trials, even when a significant preference was found, there was an intraspecific variability in the specialization of the oviposition site selection. For example, when cucumber was used with finger millet, 53.3% of the females laid 100% of their eggs on the target crop, while the rest still chose to lay a small proportion of their eggs on the banker plant. This begs the question, are American hoverflies generalist aphidophagous individuals, or is the species considered as such since it is composed of multiple specialists with varying targets? If this is the case, an artificial selection program may be applied to different isogroup lines in order to improve the level of aphid biocontrol, depending on the context [62,63,64].

## 5. Conclusions

This study showed the impact of different banker plant/focal crop systems on the oviposition preferences of the American hoverfly and its optimization in the context of biocontrol against aphids. The finger millet banker plant should be more suitable for the control of *A. gossypii* in cucumber crops, with the barley banker plant suitable for the control of *M. persicae* in pepper crops. Furthermore, this study confirms that the oviposition preference of *E. americanus* is adequate for the control of aphids in mixed cucumber/pepper crops. Of course, it is essential to confirm these oviposition preferences in the context of a commercial greenhouse in order to validate our recommendations for the choice of banker plant. It is also necessary to determine how aphid density on the focal crop will affect the female oviposition behavior, e.g., at which focal aphid density the predator will start laying eggs on the target crop, and also what is the performance of the syrphid larvae when preying upon banker and focal aphids.

## Data Availability

The data associated with this publication can be accessed on Zendodo (https://doi.org/10.5281/zenodo.7362936) (25 November 2022).

## References

[1] Rabasse J.-M., van Steenis M.J., Albajes R., Lodovica Gullino M., van Lenteren J.C., Elad Y. (1999). Biological Control of Aphids. Integrated Pest and Disease Management in Greenhouse Crops.

[2] Knapp M., Palevsky E., Rapisarda C., Gullino M.L., Albajes R., Nicot P.C. (2020). Insect and Mite Pests. Integrated Pest and Disease Management in Greenhouse Crops.

[3] Capinera J.L. (2004). Melon Aphid or Cotton Aphid, *Aphis gossypii* Glover (Insecta: Hemiptera: Aphididae): EENY-173/IN330, 11/2000. EDIS.

[4] Mahmood I., Imadi S.R., Shazadi K., Gul A., Hakeem K.R., Hakeem K.R., Akhtar M.S., Abdullah S.N.A. (2016). Effects of Pesticides on Environment. Plant, Soil and Microbes: Volume 1: Implications in Crop Science.

[5] Amorós-Jiménez R., Pineda A., Fereres A., Marcos-García M. (2012). Prey Availability and Abiotic Requirements of Immature Stages of the Aphid Predator *Sphaerophoria rueppellii*. Biol. Control.

[6] Amorós-Jiménez R., Pineda A., Fereres A., Marcos-García M.Á. (2014). Feeding Preferences of the Aphidophagous Hoverfly *Sphaerophoria rueppellii* Affect the Performance of Its Offspring. BioControl.

[7] Pekas A., De Craecker I., Boonen S., Wäckers F.L., Moerkens R. (2020). One Stone; Two Birds: Concurrent Pest Control and Pollination Services Provided by Aphidophagous Hoverflies. Biol. Control.

[8] Pineda A., Marcos-García M.A. (2008). Evaluation of Several Strategies to Increase the Residence Time of Episyrphus Balteatus (Diptera, Syrphidae) Releases in Sweet Pepper Greenhouses. Ann. Appl. Biol..

[9] Prado S.G., Jandricic S.E., Frank S.D. (2015). Ecological Interactions Affecting the Efficacy of *Aphidius colemani* in Greenhouse Crops. Insects.

[10] Jandricic S.E., Wraight S.P., Gillespie D.R., Sanderson J.P. (2016). Biological Control Outcomes Using the Generalist Aphid Predator *Aphidoletes aphidimyza* under Multi-Prey Conditions. Insects.

[11] La-Spina M., Jandricic S.E., Buitenhuis R. (2019). Short-Term Increases in Aphid Dispersal from Defensive Dropping Do Not Necessarily Affect Long-Term Biological Control by Parasitoids. J. Econ. Entomol..

[12] Fischer S., Léger A. (1997). Lutte biologique contre les pucerons du concombre en serre au moyen de plantes banques. Rev. Suisse Vitic. Arboric. Hortic..

[13] Boll R., Geria A., Marconi A., Migliore O., Salles M., Fauvergue X. (2001). Contre Les Pucerons En Serres de Concombre, Les Plantes-Relais: Une Solution de Lutte Biologique?. Phytoma Déf. Végétaux.

[14] Garzo E., Diaz B., Fereres A. (2003). Settlement Rate of *Aphis gossypii* (Hemiptera, Aphididae) and Transmission Efficiency of Cucumber Mosaic Virus in Melons Protected with Kaolin-Particle Films. Span. J. Agric. Res..

[15] Polat Akköprü E. (2018). The Effect of Some Cucumber Cultivars on the Biology of *Aphis gossypii* Glover (Hemiptera: Aphididae). Phytoparasitica.

[16] Chi B., Zhang X., Shi Q., Wang N., Liu Y. (2019). Colored Plastic Films Affect Demographic Characteristics of *Aphis gossypii* on Cucumber Plants. Int. J. Pest Manag..

[17] Alaserhat İ., Canbay A., Özdemir İ. (2021). Aphid Species, Their Natural Enemies in Vegetables from Erzincan, Turkey: First Record of the Parasitoid Wasp *Aphelinus mali* (Haldeman) Parasitizing *Lipaphis erysimi* (Kaltenbach). J. Agric. Sci..

[18] Kahia M., Nguyen T., McCune F., Naasz R., Antoun H., Fournier V. (2021). Insecticidal Effect of *Bacillus pumilus* PTB180 and *Bacillus subtilis* PTB185 Used Alone and in Combination against the Foxglove Aphid and the Melon Aphid (Hemiptera: Aphididae). Can. Entomol..

[19] Payton Miller T.L., Rebek E.J. (2018). Banker Plants for Aphid Biological Control in Greenhouses. J. Integr. Pest Manag..

[20] Frank S.D. (2010). Biological Control of Arthropod Pests Using Banker Plant Systems: Past Progress and Future Directions. Biol. Control.

[21] Huang N., Enkegaard A., Osborne L.S., Ramakers P.M.J., Messelink G.J., Pijnakker J., Murphy G. (2011). The Banker Plant Method in Biological Control. Crit. Rev. Plant Sci..

[22] Yano E. (2019). Functions of Banker Plants for Biological Control of Arthropod Pests in Protected Culture. CAB Rev. Perspect. Agric. Vet. Sci. Nutr. Nat. Resour..

[23] Rezende B.L.A., Cecílio Filho A.B., Barros Júnior A.P., Porto D.R.Q., Martins M.I.E.G. (2011). Economic Analysis of Cucumber and Lettuce Intercropping under Greenhouse in the Winter-Spring. An. Acad. Bras. Cienc..

[24] Cecílio Filho A.B., Rezende B.L.A., Barbosa J.C., Grangeiro L.C. (2011). Agronomic Efficiency of Intercropping Tomato and Lettuce. An. Acad. Bras. Cienc..

[25] Cecílio Filho A.B., Neto F.B., Rezende B.L.A., Barros Júnior A.P., de Lima J.S.S. (2015). Indices of Bio-Agroeconomic Efficiency in Intercropping Systems of Cucumber and Lettuce in Greenhouse. Aust. J. Crop Sci..

[26] Almohamad R., Verheggen F., Haubruge E. (2009). Searching and Oviposition Behavior of Aphidophagous Hoverflies (Diptera: Syrphidae): A Review. Biotechnol. Agron. Société Environ..

[27] Rodríguez-Gasol N., Alins G., Veronesi E.R., Wratten S. (2020). The Ecology of Predatory Hoverflies as Ecosystem-Service Providers in Agricultural Systems. Biol. Control.

[28] Bellefeuille Y., Fournier M., Lucas E. (2019). Evaluation of Two Potential Biological Control Agents Against the Foxglove Aphid at Low Temperatures. J. Insect Sci..

[29] Bellefeuille Y., Fournier M., Lucas E. (2021). Biological Control of the Foxglove Aphid Using a Banker Plant with *Eupeodes americanus* (Diptera: Syrphidae) in Experimental and Commercial Greenhouses. Biol. Control.

[30] Ouattara T.Y., Fournier M., Rojo S., Lucas E. (2022). Development Cycle of a Potential Biocontrol Agent: The American Hoverfly, *Eupeodes americanus*, and Comparison with the Commercial Biocontrol Agent *Aphidoletes aphidimyza*. Entomol. Exp. Appl..

[31] Hemptinne J.-L., Doucet J.-L., Petersen J.-E. (1993). Optimal Foraging by Hoverflies (Diptera: Syrphidae) and Ladybirds (Coleopteraz Coccinellidae): Mechanisms. Eur. J. Entomol..

[32] Pineda A., Morales I., Marcos-García M.A., Fereres A. (2007). Oviposition Avoidance of Parasitized Aphid Colonies by the Syrphid Predator *Episyrphus balteatus* Mediated by Different Cues. Biol. Control.

[33] Almohamad R., Verheggen F.J., Francis F., Haubruge E. (2010). Intraguild Interactions between the Predatory Hoverfly *Episyrphus balteatus* (Diptera: Syrphidae) and the Asian Ladybird, *Harmonia axyridis* (Coleoptera: Coccinellidae): Effect of Larval Tracks. Eur. J. Entomol..

[34] Amiri-Jami A.R., Sadeghi H., Gilbert F., Moravvej G., Asoodeh A. (2016). Oviposition Preference of Aphidophagous Hoverflies toward Oviposition Site Quality: The Presence of Intra- and Interspecific Competitor, Glucosinolate Content, and Prey Species. J. Asia-Pac. Entomol..

[35] Dunn L., Lequerica M., Reid C.R., Latty T. (2020). Dual Ecosystem Services of Syrphid Flies (Diptera: Syrphidae): Pollinators and Biological Control Agents. Pest Manag. Sci..

[36] Sadeghi H., Gilbert F. (2000). Aphid Suitability and Its Relationship to Oviposition Preference in Predatory Hoverflies. J. Anim. Ecol..

[37] Almohamad R., Verheggen F.J., Francis F., Haubruge E. (2007). Predatory Hoverflies Select Their Oviposition Site According to Aphid Host Plant and Aphid Species. Entomol. Exp. Appl..

[38] Vanhaelen N., Haubruge E., Gaspar C., Francis F. (2001). Oviposition Preferences of *Episyrphus balteatus*. Meded. Rijksuniv. Te Gent Fak. Van Landbouwkd. En Toegepaste Biol. Wet..

[39] Messelink G.J., Calvo F.J., Marín F., Janssen D., Gullino M.L., Albajes R., Nicot P.C. (2020). Cucurbits. Integrated Pest and Disease Management in Greenhouse Crops.

[40] Messelink G.J., Labbé R., Marchand G., Tavella L., Gullino M.L., Albajes R., Nicot P.C. (2020). Sweet Pepper. Integrated Pest and Disease Management in Greenhouse Crops.

[41] Jacobson R.J., Croft P. (1998). Strategies for the Control of *Aphis gossypii* Glover (Hom.: Aphididae) with *Aphidius colemani* Viereck (Hym.: Braconidae) in Protected Cucumbers. Biocontrol Sci. Technol..

[42] Goh H.G., Kim J.H., Han M.W. (2001). Application of *Aphidius colemani* Viereck for Control of the Aphid in Greenhouse. J. Asia-Pac. Entomol..

[43] Branquart E., Hemptinne J.-L. (2000). Development of Ovaries, Allometry of Reproductive Traits and Fecundity of *Episyrphus balteatus* (Diptera: Syrphidae). Eur. J. Entomol..

[44] Orengo-Green J.J., Casas J.L., Marcos-García M.Á. (2022). Effect of Abiotic Climatic Factors on the Gonadal Maturation of the Biocontrol Agent *Sphaerophoria rueppellii* (Wiedemann, 1830) (Diptera: Syrphidae). Insects.

[45] Higashida K., Yano E., Nishikawa S., Ono S., Okuno N., Sakaguchi T. (2016). Reproduction and Oviposition Selection by *Aphidoletes aphidimyza* (Diptera: Cecidomyiidae) on the Banker Plants with Alternative Prey Aphids or Crop Plants with Pest Aphids. Appl. Entomol. Zool..

[46] Verheggen F.J., Capella Q., Schwartzberg E.G., Voigt D., Haubruge E. (2009). Tomato-Aphid-Hoverfly: A Tritrophic Interaction Incompatible for Pest Management. Arthropod Plant Interact..

[47] Sobhani M., Madadi H., Gharali B. (2013). Host Plant Effect on Functional Response and Consumption Rate of *Episyrphus balteatus* (Diptera: Syrphidae) Feeding on Different Densities of *Aphis gossypii* (Hemiptera: Aphididae). J. Crop Prot..

[48] Riddick E.W., Simmons A.M. (2014). Do Plant Trichomes Cause More Harm than Good to Predatory Insects?. Pest Manag. Sci..

[49] Sadeghi H. (2002). The Relationship Between Oviposition Preference and Larval Performance in an Aphidophagous Hover Fly, *Syrphus ribesii* L. (Diptera: Syrphidae). J. Agric. Sci. Technol..

[50] Chandler A.E.F. (1969). Locomotory Behaviour of First Instar Larvae of Aphidophagous Syrphidae (Diptera) after Contact with Aphids. Anim. Behav..

[51] Scholz D., Poehling H.-M. (2000). Oviposition Site Selection of *Episyrphus balteatus*. Entomol. Exp. Appl..

[52] Ambrosino M.D., Jepson P.C., Luna J.M. (2007). Hoverfly Oviposition Response to Aphids in Broccoli Fields. Entomol. Exp. Appl..

[53] Widstrom N.W., Mcmillian W.W., Wiseman B.R. (1979). Ovipositional Preference of the Corn Earworm and the Development of Trichomes on Two Exotic Corn Selections. Environ. Entomol..

[54] Durbey S.L., Sarup P. (1982). Morphological Characters—Development and Density of Trichomes on Varied Maize Germplasms in Relation to Preferential Oviposition by the Stalk Borer, *Chilo partellus* (Swinhoe). J. Entomol. Res..

[55] Kumar H. (1992). Inhibition of Ovipositional Responses of *Chilo partellus* (Lepidoptera: Pyralidae) by the Trichomes on the Lower Leaf Surface of a Maize Cultivar. J. Econ. Entomol..

[56] Cortesero A.M., Stapel J.O., Lewis W.J. (2000). Understanding and Manipulating Plant Attributes to Enhance Biological Control. Biol. Control.

[57] Budenberg W.J., Powell W. (1992). The Role of Honeydew as an Ovipositional Stimulant for Two Species of Syrphids. Entomol. Exp. Appl..

[58] Leroy P.D., Almohamad R., Attia S., Capella Q., Verheggen F.J., Haubruge E., Francis F. (2014). Aphid Honeydew: An Arrestant and a Contact Kairomone for *Episyrphus balteatus* (Diptera: Syrphidae) Larvae and Adults. Eur. J. Entomol..

[59] Sutherland J.P., Sullivan M.S., Poppy G.M. (2001). Oviposition Behaviour and Host Colony Size Discrimination in *Episyrphus balteatus* (Diptera: Syrphidae). Bull. Entomol. Res..

[60] Almohamad R., Verheggen F., Francis F., Haubruge E. (2006). Evaluation of Hoverfly *Episyrphus balteatus* De Geer (Diptera: Syrphidae) Oviposition Behaviour toward Aphid-Infested Plants Using a Leaf Disc System. Commun. Agric. Appl. Biol. Sci..

[61] Nelson E.H., Hogg B.N., Mills N.J., Daane K.M. (2012). Syrphid Flies Suppress Lettuce Aphids. BioControl.

[62] Pu D., Zheng Z., Liu H., Wang X., Wu X., Chen Y., Deng J., Chen X., Li Y. (2019). Development and Reproduction of the Hoverfly *Eupeodes corollae* (Diptera: Syrphidae). SDRP J. Earth Sci. Environ. Stud..

[63] Parajulee M.N. (2007). Influence of Constant Temperatures on Life History Parameters of the Cotton Aphid, *Aphis gossypii*, Infesting Cotton. Environ. Entomol..

[64] Satar S., Kersting U., Uygun N. (2008). Effect of Temperature on Population Parameters of *Aphis gossypii* Glover and *Myzus persicae* (Sulzer) (Homoptera: Aphididae) on Pepper. J. Plant Dis. Prot..

